# Peripheral nerve injury induces adult brain neurogenesis and remodelling

**DOI:** 10.1111/jcmm.12965

**Published:** 2016-09-24

**Authors:** Gabriel Rusanescu, Jianren Mao

**Affiliations:** ^1^ MGH Center for Translational Pain ResearchDepartment of AnesthesiaCritical Care and Pain MedicineMassachusetts General HospitalHarvard Medical SchoolBostonMAUSA

**Keywords:** structural plasticity, adult neurogenesis, chronic pain, cerebellum, cross‐modal perception, proprioception, Notch3, Purkinje neurons

## Abstract

Unilateral peripheral nerve chronic constriction injury (CCI) has been widely used as a research model of human neuropathic pain. Recently, CCI has been shown to induce spinal cord adult neurogenesis, which may contribute to the chronic increase in nociceptive sensitivity. Here, we show that CCI also induces rapid and profound asymmetrical anatomical rearrangements in the adult rodent cerebellum and pons. This remodelling occurs throughout the hindbrain, and in addition to regions involved in pain processing, also affects other sensory modalities. We demonstrate that these anatomical changes, partially reversible in the long term, result from adult neurogenesis. Neurogenic markers Mash1, Ngn2, doublecortin and Notch3 are widely expressed in the rodent cerebellum and pons, both under normal and injured conditions. CCI‐induced hindbrain structural plasticity is absent in Notch3 knockout mice, a strain with impaired neuronal differentiation, demonstrating its dependence on adult neurogenesis. Grey matter and white matter structural changes in human brain, as a result of pain, injury or learned behaviours have been previously detected using non‐invasive neuroimaging techniques. Because neurogenesis‐mediated structural plasticity is thought to be restricted to the hippocampus and the subventricular zone, such anatomical rearrangements in other parts of the brain have been thought to result from neuronal plasticity or glial hypertrophy. Our findings suggest the presence of extensive neurogenesis‐based structural plasticity in the adult mammalian brain, which may maintain a memory of basal sensory levels, and act as an adaptive mechanism to changes in sensory inputs.

## Introduction

Recent non‐invasive techniques have suggested that the brain undergoes significant structural plasticity as a result of learning 1, 2, 3, 4, injury 5, 6 or pain 7, 8. These structural changes involve macroscopic alterations in grey matter and/or white matter (WM) volumes as well as density. Since adult neurogenesis is known to occur in the brain primarily in two niches, the dentate gyrus (DG) and the subventricular zone (SVZ) 9, 10, 11, 12, 13, the structural changes that occur in other parts of the brain have been credited to neuronal plasticity and glial hypertrophy. However, experimental data suggest a substantial discrepancy between the relatively large anatomical changes induced by learning or injury, and the microscopic scale of neuronal plasticity. Although newly generated neurons have been also observed in other parts of the adult mammalian brain 14, 15, 16, the presence of neurogenesis outside of the DG and the SVZ has been disputed 17. For example, the cerebellum has been also involved in both pain 18, 19 and cognitive 20, 21, 22, 23 processing. However, the presence of adult cerebellar neurogenesis in mammals has been rejected 24, 25, although newly generated neurons 26 and morphometric changes 8, 27 have been found. The adult neurogenesis observed in rabbit cerebellum has been thought to constitute an exception relative to other mammals 28. In general, the prevailing view since the early days of ^3^H autoradiography has been that cerebellar neurogenesis is negligible in the adult, and the cerebellum has been considered to be one of the most static parts of the adult mammalian brain 29.

We have previously shown that chronic constriction injury of the sciatic nerve (CCI) in rodents, a commonly used model of chronic neuropathic pain 30, induces substantial adult spinal cord neurogenesis 31. Concurrently with the brain morphometric changes induced by pain, this raised the possibility that a similar neurogenic process may also be elicited in the pain processing centres in the brain. Although adult neurogenesis is commonly thought to be restricted to the DG and SVZ, it has been recently suggested that the detection of proliferating neuronal progenitor cells (NPCs) may be limited by the sensitivity of the experimental methods used 32. Therefore, we investigated the possibility that the brain structural changes associated with chronic pain may also involve adult neurogenesis in addition to well‐established neuronal plasticity mechanisms.

As a result of pain avoidance, CCI induces in rodents significant chronic posture changes, which involve regulation by the proprioceptive and vestibular sensory systems and the motor system. These systems intersect in the cerebellum, which acts as an integrator and comparator of ascending and descending nociceptive, proprioceptive and motor information, representing a logical target for the investigation of pain‐induced structural plasticity. The nociceptive information that reaches the ipsilateral spinal cord dorsal horn is for the most part transmitted *via* second‐order axons, which cross to the contralateral side and reach the brain as the spino‐thalamic tract. Nociceptive information is then transmitted to the somatosensory cortex contralateral to the injury site. Descending cortico‐cerebellar pathways deliver to the cerebellum information from several brain regions, including the somatosensory, motor and visual cortices, the prefrontal and temporal lobes, the hippocampus and the hypothalamus, *via* two major routes, the pontine nuclei and the inferior olives, likely inducing a conscious change in posture to the side contralateral to injury. This posture change is compared with ascending proprioceptive information from muscle spindles and Golgi tendon organs, relayed through the ascending dorsal and ventral spinocerebellar tracts. These connect to the cerebellum through the inferior olives and the inferior and superior cerebellar peduncles, crossing back to the side ipsilateral to the movement activity (and contralateral to injury). Vestibular information is relayed to the cerebellum through the vestibular nuclei (vn), also on the ipsilateral side.

In addition to the indirect, descending cortico‐cerebellar pain inputs, the pontine nuclei and the inferior olives may also directly relay ascending nociceptive information to the cerebellum, through a spino‐pontocerebellar path and a spino‐olivocerebellar path, respectively, which are less understood 18. Electrophysiological evidence suggests that C‐fibre stimulation activates the mossy fibre input from the pontine nuclei 33, and that both A‐fibre and C‐fibre stimulation activates the climbing fibres 34, which transmit information from the inferior olive to the cerebellum. In addition, the zona incerta and the periaqueductal grey, two brain regions that deliver major inputs to the inferior olives, are known to relay both ascending nociceptive information 35, 36, 37, as well as descending cortical signals.

To test the possibility that adult neurogenesis may be involved in brain structural changes induced by pain, we used the same CCI model used previously to demonstrate spinal cord neurogenesis and structural plasticity 31. We find that within 6 weeks, CCI induces major anatomical rearrangements in the rodent cerebellum and pons, correlating with the time frame of spinal cord neurogenesis. This extensive remodelling correlated with the functional transfer of proprioceptive and vestibular modalities to the contralateral side, as reflected in behavioural changes. The large‐scale structural change was not limited to centres for nociceptive, vestibular or motor processing but was generalized throughout the entire cerebellum and pons, including areas involved in auditory and gustatory sensory modalities that are not expected to be influenced by pain. Using well‐described markers for sequential stages of neuronal differentiation, we demonstrate that this profound and long‐lasting hindbrain remodelling involves new, adult‐generated neurons. These findings suggest the possibility that the mammalian brain could undergo neuronal regeneration and remodelling in other areas, in addition to currently recognized neurogenic regions. They also point to a potential contribution of neurogenesis to a cross‐modal sensory integration of nociception with other sensory systems.

## Materials and methods

### Animals and surgery

Two‐month‐old SD male rats (Charles River Labs, Wilmington, MA, USA) and C57B/6J wild‐type (WT) and Notch3 KO mice (The Jackson Labs, Bar Harbor, ME, USA) were housed under standard conditions with free access to food and water. Animals were subjected to unilateral constriction injury of the right sciatic nerve (CCI) 30 under sodium pentobarbital anaesthesia (50 mg/kg, intraperitoneally). Unilateral CCI was performed by placing four chromic gut ligatures around the right sciatic nerve. Animals not subjected to surgery (naïve) and animals subjected to sham surgery, without placing sciatic nerve ligatures, were used as controls. Five animals per condition were killed at 0, 6 and 52 weeks after CCI by perfusion‐fixation for immunofluorescence analysis. Experimental protocols were approved by the IACUC Committee at Massachusetts General Hospital.

### Immunofluorescence

Fixed rat and mouse brains were frozen in Tissue‐Tek OCT and cryo‐sectioned into transverse 35‐μm‐thick slices. Slices were permeabilized for 2 hrs (3% bovine serum albumin and 0.2% Triton in PBS), then incubated overnight at 4°C with primary antibodies, washed in PBS and incubated 1.5 hrs in Cy3‐ and FITC‐linked secondary antibodies (Jackson Immuno‐research, West Grove, PA, USA). Primary antibodies included doublecortin (DCX), Mash1, Ngn2, calretinin (CR; Santa Cruz, Dallas, TX, USA), GFAP (BD Biosciences, San Jose, CA, USA), calbindin (CB) and NeuN (Millipore, Billerica, MA, USA). Antibody specificity was attested by published reports and manufacturer's data or tested by double labelling. Slices were mounted on slides and imaged using an Olympus‐80 fluorescence microscope equipped with FITC and Cy3 filters. Bleed‐through was minimized by dual scanning on two different FITC‐Cy3 filter sets with slightly different bandpass windows.

### EdU labelling

Six‐week‐old mice (*n* = 5) were subjected to CCI, then 5 weeks later were injected twice daily for 3 days with 5‐Ethynyl‐2‐deoxyuridine (EdU; 4 mg/kg i.p., Jena Bioscience, Jena, Germany) 38. Four days after last injection, the mice were killed by perfusion‐fixation under anaesthesia. Brain slices were permeabilized 30 min. in 0.2% Triton/TBS then incubated for 15 min. at room temperature with azide‐fluorescein (Jena Bioscience) and 1 mM Cu^+^. The slices were then washed and subjected to immunostaining as above. The slices were mounted on slides using mounting medium (Vectashield, Vector Labs, Burlingame, CA, USA) and imaged by immunofluorescence.

### Statistical analysis

Immunofluorescence was quantified for hindbrain areas indicated in Figure 2 using NIH Image J. Measurements were averaged over three adjacent slices per animal, for five animals per treatment (Control and CCI), attempting to include slices from the same brain location in each animal. The statistical significance of differences between area measurements corresponding to Control *versus* CCI‐Contralateral, and Control *versus* CCI‐Ipsilateral was evaluated by paired two‐tailed *t*‐tests. Animals were randomly assigned to each treatment group.

## Results

### CCI induces hindbrain remodelling

**Figure 1:**
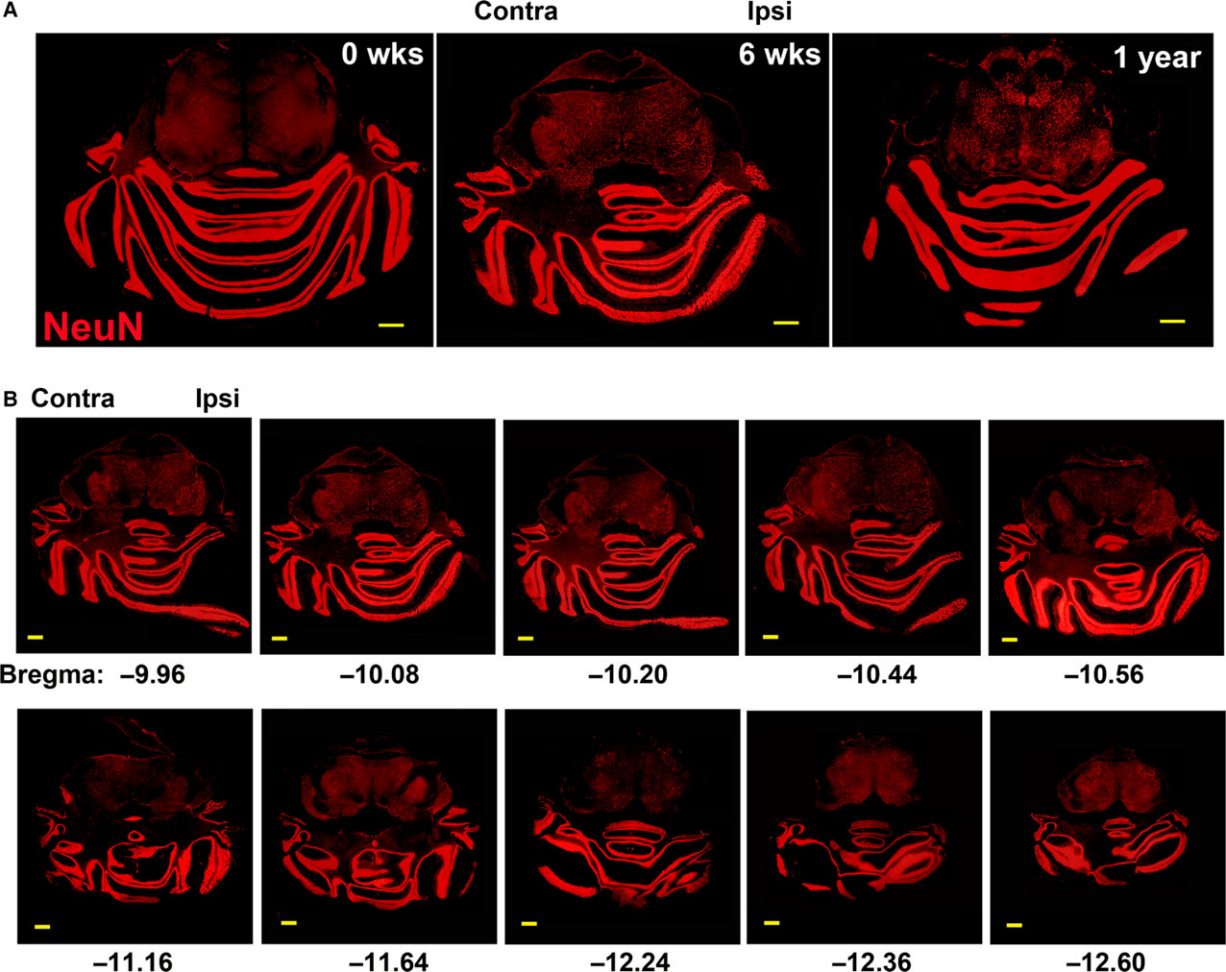
CCI induces rat hindbrain anatomical remodelling. (**A**) Immunofluorescence analysis of coronal sections through adult rat cerebellum and pons, before (Control), 6 weeks and 1 year post‐CCI, stained for neuronal marker NeuN. (**B**) Sequential sections of rat hindbrain 6 weeks post‐CCI, stained for neuronal marker NeuN, show comprehensive contralateral–ipsilateral anatomical reorganization. Bregma values indicate section position. Scale bars represent 1 mm. Contra: contralateral; Ipsi: ipsilateral.

Non‐invasive imaging data have suggested that chronic pain can lead to anatomical rearrangements in the hindbrain 8, 27, 39. Therefore, we investigated whether such changes are also noticeable after unilateral chronic constriction injury of the sciatic nerve (CCI), a widely used rodent model of chronic neuropathic pain. We find that, within 6 weeks post CCI, rapid and significant anatomical remodelling was induced in the rat cerebellum and pons, as revealed by staining for neuronal marker NeuN. This time‐point was chosen based on a preceding spinal cord study 31, as optimal for the observation of neurogenesis‐mediated structural plasticity. The remodelling resulted in striking anatomical differences between the contralateral and ipsilateral sides relative to injury (Fig. 1A). These changes were specifically present in the cerebellum and pons, but not obvious in the rest of the brain. The remodelling was only partially reversible in the long term, remaining visible for at least 1 year post‐CCI. The anatomical remodelling was present throughout the hindbrain, reflected in representative anterior to posterior saggital brain sections (Fig. 1B). The allodynia and hyperalgesia associated with this time‐point post‐CCI have been described previously 31 and will not be repeated here.

**Figure 2:**
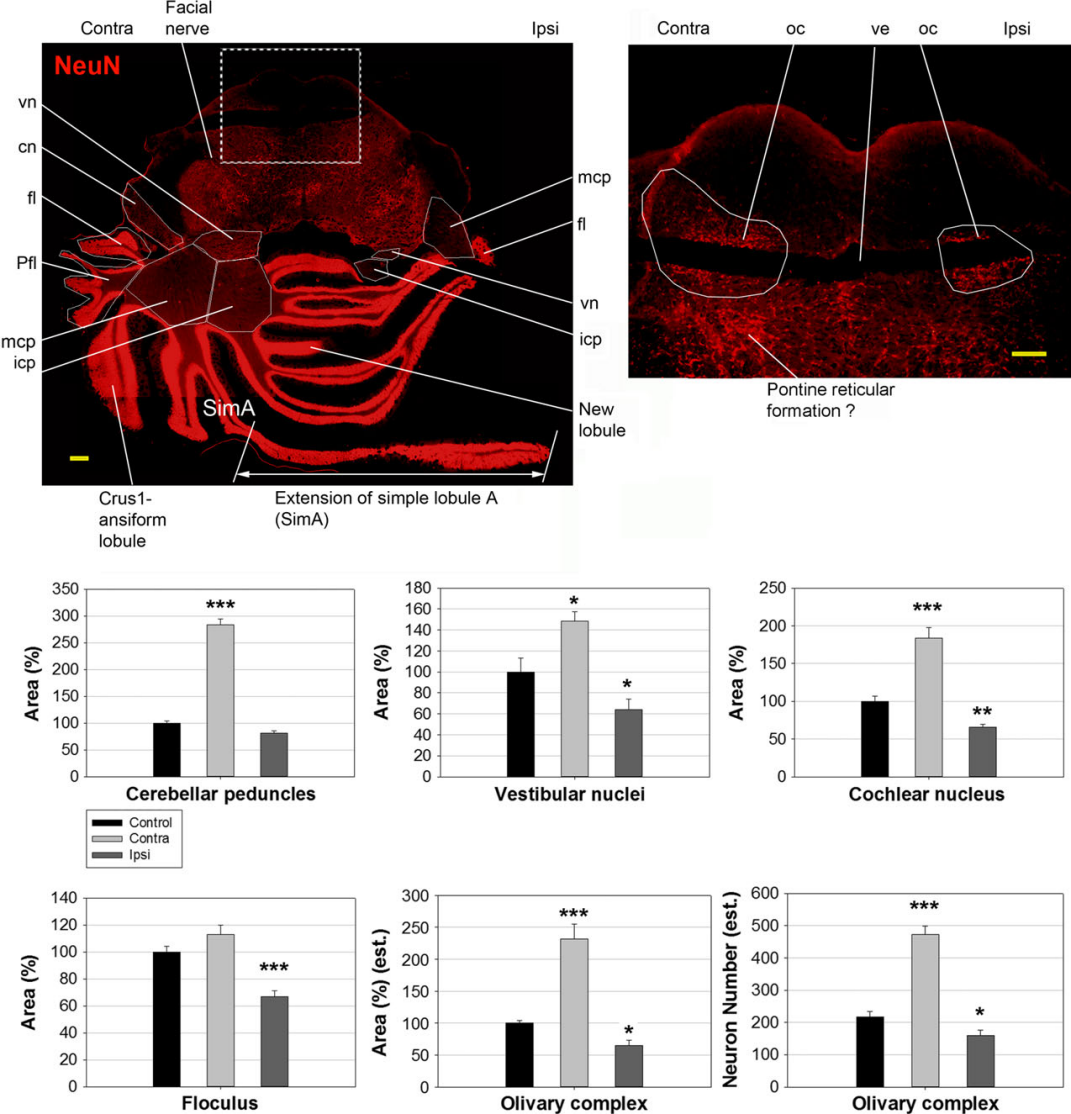
Structural plasticity in rat brain, 6 weeks post CCI. Some major remodelled structures are outlined, and their changes in size are illustrated in graphs below. Scale bars represent 1 mm (figure), 200 μm (inset). vn: vestibular nucleus; cn: cochlear nucleus; fl: floculus; Pfl: parafloculus; mcp: middle cerebellar peduncle; icp: inferior cerebellar peduncle; oc: olivary complex; ve: ventricular extension; Contra: contralateral; Ipsi: ipsilateral. Statistical significance: “*” corresponds to 0.01<p<0.05, “**” corresponds to 0.001<p<0.01, “***” corresponds to p<0.001.

The hindbrain remodelling included an expansion of vestibular and proprioceptive structures contralateral to CCI and a contraction or elimination of their ipsilateral equivalents, corresponding to the transfer of motor and sensory modalities from the injured to the healthy leg (Fig. 2). These anatomical changes were associated with obvious posture changes, which are well known and described in literature, for example, animals refrained from using the injured leg, concentrating their motor activities and balance on the contralateral, uninjured leg. The anatomical reorganization included the middle and inferior cerebellar peduncles, the vestibular nuclei (vn), the floculus and potentially the pontine reticular formation, which were increased in size contralaterally and reduced ipsilaterally, while the parafloculus, the ansiform lobule and simple lobule A were completely eliminated ipsilaterally. The contralateral simple lobule A appeared fused with an extension similar to a cerebellar lobule, but detached from the ipsilateral peduncle. Regions related to other sensory modalities, such as the cochlear nuclei (cn), the facial nerve and the olivary complex (oc) also underwent sizeable changes. In addition, several new structures appeared that do not exist in control animals, such as a new ventricular extension throughout the length of the ventral pons (shown magnified), additional cerebellar lobules connected to the contralateral peduncles, as well as the extension of contralateral lobule A. This increased to seven, the number of cerebellar lobules connected to the contralateral peduncles, while the number of lobules connected ipsilaterally was reduced to two. Changes in size after CCI of the most representative hindbrain regions are shown, relative to control animals, including the statistical significance of changes. Changes in area and neuron numbers for the oc should be considered only as an estimate, given the difficulty of establishing exact boundaries for each anatomical structure in the absence of a physiological correlation. In addition, CCI significantly distorts brain anatomy, making the matching of sections from CCI and Control animals difficult. Some brain structures are displaced from their normal location, therefore a more exact quantification can be only obtained through a complete brain sectioning and volumetric quantification involving hundreds of slices per brain.

The hindbrain structures that undergo the most significant structural plasticity following CCI concurrently process proprioceptive, motor and pain information, therefore it is difficult to separate the roles of posture changes in inducing structural plasticity from the role of pain. For example, the middle cerebellar peduncles, which undergo significant remodelling, convey information to the cerebellum from the pontine reticular formation and the pontine nuclei, which receive both ascending nociceptive information and descending cortical input. The fact that the somatosensory cortex, directly innervated by the ascending pain pathway, does not show any detectable changes may suggest that the observed hindbrain remodelling is indirectly caused by pain, by inducing chronic changes in proprioceptive, vestibular and motor inputs through descending cortico‐cerebellar pathways. However, ascending nociceptive direct inputs to the cerebellum, as described above, are also likely to have a contribution to the observed anatomical changes.

Since mature neurons, integrated in signalling networks, are unlikely to migrate large distances from the ipsilateral to the contralateral side of the brain, a reasonable explanation for this remodelling is that new neurons are generated contralaterally to CCI, to compensate for the marked increase in locomotor function on that side. It is interesting to note that, while pre‐existing cerebellar lobules show two separate, parallel layers of NeuN+ neurons, corresponding to the granule cell layers (GL; nuclear layers), separated by WM, the newly generated lobule shows only a single, wider layer of neurons. This may reflect an initial bulk generation of new neurons, similar to embryonic development, which only later becomes organized into distinct GLs.

### CCI‐induced hindbrain remodelling is associated with adult‐generated Purkinje neurons

**Figure 3:**
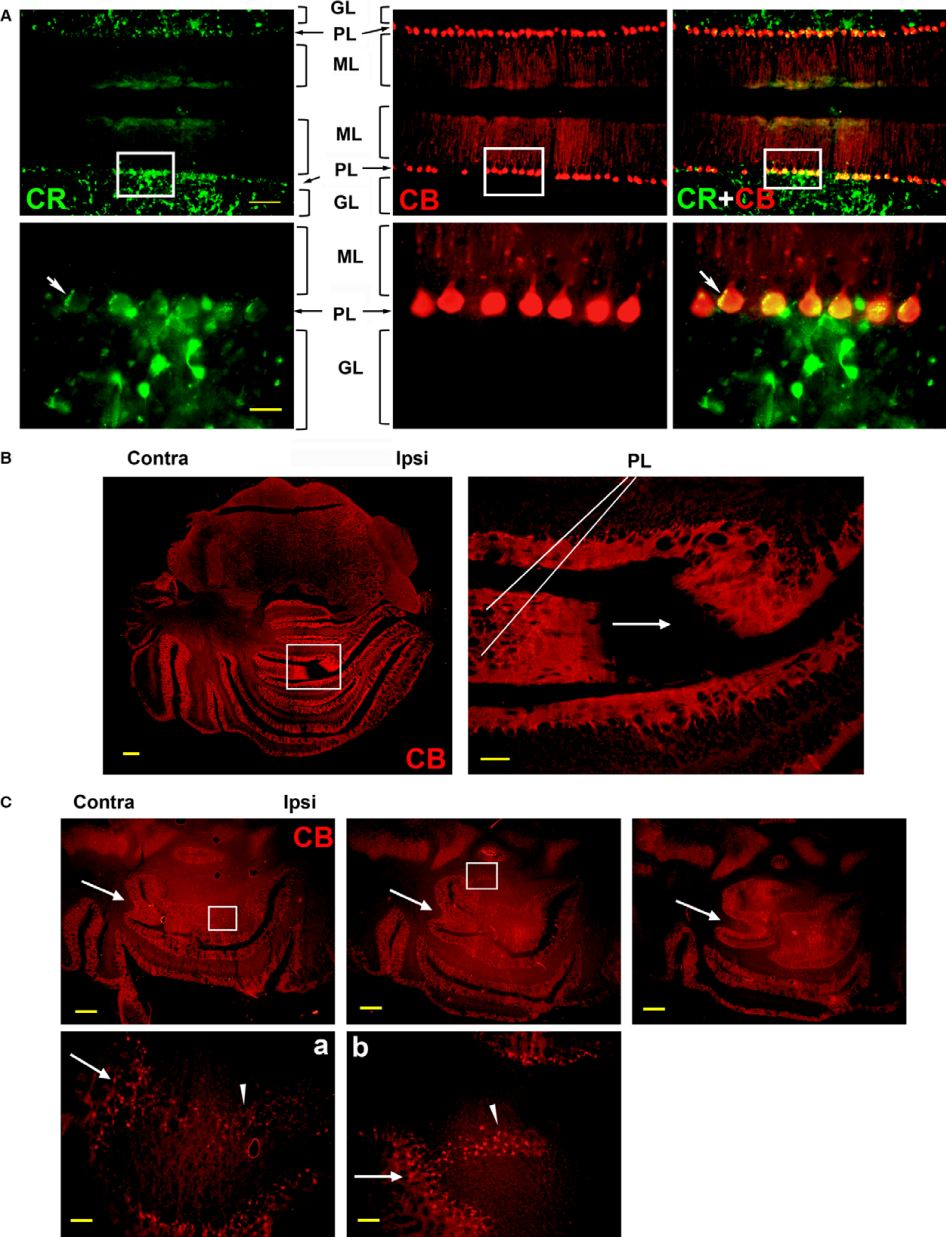
Hindbrain structural remodelling is associated with adult‐generated Purkinje neurons. (**A**) Immunofluorescence imaging of a cerebellar section from a control rat, showing that calbindin (CB), a marker for a subset of GABA‐ergic neurons, is specific for Purkinje neurons in the cerebellum. Neurons in the PL express CB, but not calretinin (CR), a marker for a different subtype of GABA‐ergic neurons. In contrast, neurons in the granule cell layer (GL) stain for CR but not for CB. Synapses of CR+ neurons on CB+ Purkinje neuron bodies are visible (detail, arrowheads). The CB‐stained typical dendritic trees of the Purkinje neurons are visible in the molecular layer (ML). The white frame outlines the position of detail panels. (**B**) Coronal section through adult rat hindbrain, 6 weeks post CCI, stained for CB, shows the asymmetric growth of a new cerebellar lobule, from the contralateral towards the ipsilateral side. Inset shows intense CB staining at the lobule growth front, suggesting that lobule growth is associated with the production of new Purkinje neurons. Arrow indicates contralateral to ipsilateral direction of growth. PL: Purkinje layer. (**C**) Sequential sections (Bregma −10.8 to −10.9) of rat hindbrain, 6 weeks post CCI, illustrate the reorganization of cerebellar lobules and the progressive growth of a new lobule. Invaginations (arrows) form from the contralateral side to initiate new lobules. New Purkinje neurons accumulate at specific sites (details **a** and **b**, arrows) on the existing mono‐cellular PL, and create loose neuron bridges through the white matter from the contralateral side (arrowheads). Scale bars: 200 μm (**A**), 20 μm (**A**‐detail), 1 mm (**B**,** C**), 200 μm (**B**‐detail), 100 μm (**Ca**,** Cb**).

A typical feature of the cerebellum are the Purkinje neurons, large, inhibitory GABAergic neurons which relay the motor coordination output of the cerebellum. These neurons are normally arranged in characteristic monocellular layers (Purkinje layer – PL) that line the cerebellar lobules, at the interface of the GL and molecular layer (ML). Purkinje neurons are recognizable by their CB expression 40 (Fig. 3A, red). Calbindin expression appears to be specific for Purkinje neurons, while some of the GL neurons are CR‐positive. Figure 3A details also show synapses of CR+ neurons on CB+ Purkinje cells (detail, white arrows). We found that 6 weeks post CCI, CB expression in the rat hindbrain shows an anatomical reorganization pattern very similar to that observed for NeuN expression, including the emergence of a new cerebellar lobule, growing predominantly from the contralateral side (Fig. 3B and 3B‐inset, arrow shows direction of growth; compare to Fig. 2). However, unlike NeuN staining, which revealed the absence of a layer organization in the new lobule (Fig. 2), CB was expressed for the most part in two distinct Purkinje cell layers, suggesting that Purkinje neurons lead the cellular organization of the new lobule (Fig. 3B‐inset, PL). The tips of the two growing arms of the new lobule show abundant CB expression, suggesting that Purkinje neurons are generated at the growth front of the new lobule and contribute to its cellular organization. Figure S1 shows a higher magnification of this image, where a cluster of CB+ cells is clearly visible at the tip of the growing lobule. Further from the tip these cells become organized in two layers typical for the PLs observed in the neighbouring lobules.

The reorganization of the PLs after CCI is clearly visible in Figure 3C. Sequential brain sections show a growing invagination of the existing PL (Fig. 3C, arrows), which appears to grow into a new lobule from the contralateral side. In addition, the formation of new lobules was also initiated by local accumulations of Purkinje neurons, which represent anomalies from the normally monocellular PL (Fig. 3C, a and b, arrows, compare to Fig. 3A). The Purkinje neurons from these accumulations then migrate into the WM outlining the position of the future lobule (Fig. 3C, a and b, arrowheads). The contralateral accumulations of Purkinje neurons suggest that these neurons were generated in the adult, as a result of increased functional demand on that side, and could not have been redistributed from the ipsilateral side.

### New cerebellar lobules form through adult neurogenesis

**Figure 4:**
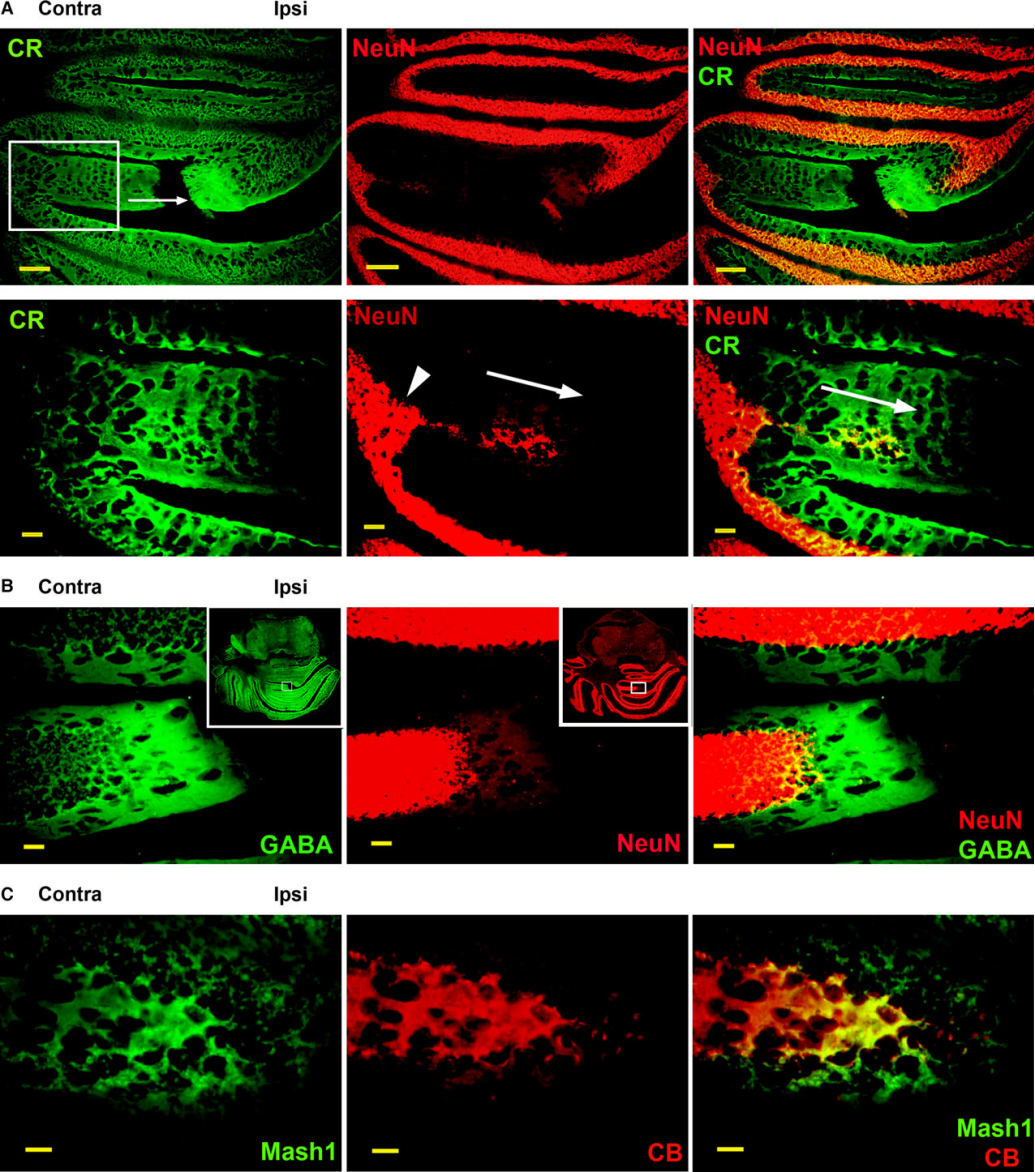
New cerebellar lobules form in rat brain 6 weeks post CCI, mediated by adult neurogenesis. (**A**) Calretinin (CR) staining shows a new cerebellar lobule growing asymmetrically from the contralateral side (arrow). Very few mature neurons, indicated by NeuN staining, are present in the new lobule, suggesting that newly generated CR+ immature neurons precede the formation of mature NeuN+ neurons. The NeuN+ granule cell layer becomes enlarged at the site of the formation of a new cerebellar lobule (inset, arrowhead), as the maturation of newly generated neurons progresses through the new lobule, showing delayed NeuN expression (inset, arrows). (**B**) GABA release at the growing tip of a new lobule precedes the formation of NeuN+ mature neurons. Inset shows detail position. (**C**) Purkinje neurons (identified by CB staining) co‐express neurogenic marker Mash1 (green) at the tip of a new lobule, suggesting that they are newly generated. Scale bars: 500 μm (**A**), 100 μm (**A**‐detail, **B**), 50 μm (**C**).

We further analysed the possibility that adult neurogenesis could play a role in the nociception‐induced brain remodelling. Calretinin, a marker for a subset of GABAergic neurons, can be used as an indicator of immature neurons 38, 41, which may suggest the presence of adult neurogenesis. In a brain section adjacent to the section shown in Figure 3B, we found that CR was expressed throughout the cerebellar lobules’ grey matter, including in the contralateral and ipsilateral arms of the new lobule, similar to CB expression (Fig. 4A). In contrast to Control animals (shown in Fig. 3A), CR staining in CCI animals was more diffuse, especially in the new lobule. NeuN staining, indicative of mature neurons, was present in only a few cells near the midline of the new lobule. At the position where the new lobule was emerging, a thickening of the pre‐existing GL was clearly visible (Fig. 4A, inset, arrowhead). The marked difference between CR and NeuN staining of the new lobule suggests that it contains predominantly immature neurons, which stain for CR, but not NeuN. In contrast to CB, CR expression was only increased at the tip of the ipsilateral, but not contralateral arm, suggesting that CB+ Purkinje neuron precursors predominantly lead the formation of a new lobule (compare to Fig. 3B).

The increased CB expression at the tip of the growing lobule, shown in Figure 3B, correlates with a similar increase in GABA staining (Fig. 4B), supporting the notion that this area contains a high concentration of GABAergic neurons, likely indicative of Purkinje neurons. GABA staining appears to be more intense in areas where NeuN expression is absent, possibly because GABA elicits an excitatory response in immature neurons 42, 43, leading to additional GABA release. In addition, GABA also acts as a trophic factor that regulates neurogenesis 44, 45.

### Expression of neurogenic markers in rodent hindbrain

**Figure 5:**
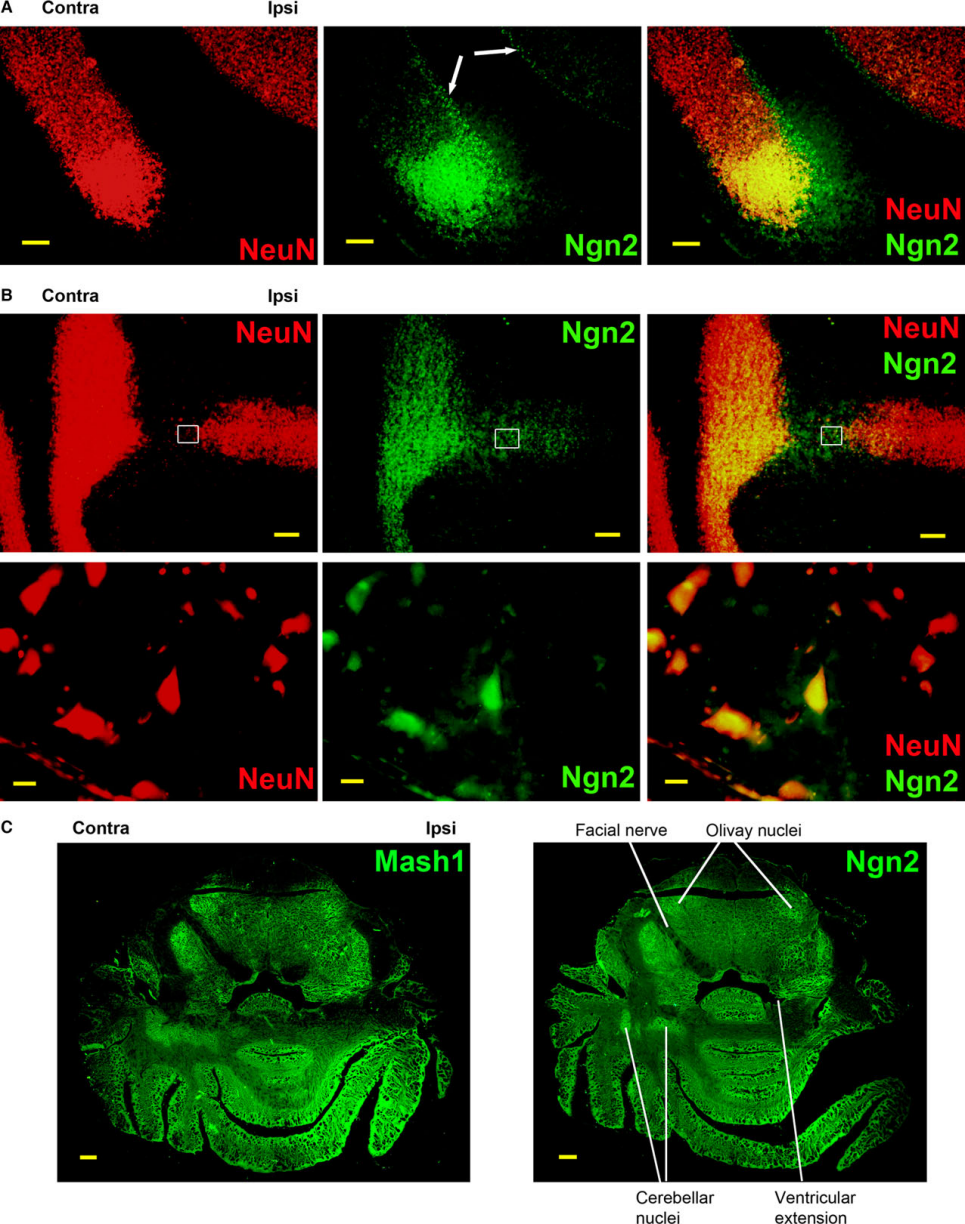
Neurogenic markers are widely expressed 6 weeks post CCI in the adult rat cerebellum. (**A**) The tip of a new lobule shows neuronal marker NeuN overlapping with neurogenic marker Ngn2, suggesting the presence of immature neurons. In both growing and existing lobules, the granule cell layers indicated by NeuN staining are lined by monocellular Ngn2+ layers, corresponding to Purkinje layers (arrows). (**B**) The new cerebellar lobule also adds new neurons at its trailing end. Colocalization of NeuN and Ngn2 in the area of new lobule growth indicates the presence of immature neurons (detail). (**C**) Altered brain structures, such as the olivary and cerebellar nuclei, only weakly visible with NeuN staining, are underlined by neurogenic markers Mash1 and Ngn2, suggesting these areas have high neurogenic activity. The ipsilateral facial nerve is shifted to a posterior position and not visible in the same section as the contralateral facial nerve. Scale bars: 500 μm (**A**), 1 mm (**B**,** C**), 10 μm (**B**‐detail).

The idea that the growth of a new cerebellar lobule is the result of adult neurogenesis was further supported by the expression patterns of Mash1 and Ngn2, markers specific for NPCs 46, 47. Mash1 is predominantly expressed at the tip of the growing lobule, overlapping with CB expression (Fig. 4C). Similar to Mash1, Ngn2 expression is significantly increased at the growing tip of cerebellar lobules, and is also expressed in a monocellular layer at the edge of the GL, overlapping with the PL (Fig. 5A, arrows). At its junction with the cerebellar peduncle, the newly developing cerebellar lobule shows a region with few mature neurons but a large number of Ngn2‐positive NPCs, suggesting that the new lobule also grows by adding new neurons at its trailing end that connects with the peduncle (Fig. 5B). Although Ngn2 expression is transient and thought to be restricted to neural progenitor cells, Ngn2 and NeuN expression may briefly overlap during the transition from immature to mature neurons. Therefore, the co‐localization of NeuN and Ngn2 expression indicates newly generated, immature neurons (Fig. 5B, detail). The macroscopic expression of Mash1 and Ngn2 also indicates other hindbrain regions where adult neurogenesis is most active during CCI‐induced remodelling (Fig. 5C). In addition to the brain changes outlined by NeuN and CB staining, Mash1 and Ngn2 highlighted additional asymmetrically modified neurogenic regions, including the cerebellar, olivary and vestibular nuclei, which expanded contralaterally following CCI. These areas were only weakly detected by NeuN staining (compare with Fig. 2), suggesting the predominance of neuron progenitors *versus* mature neurons at these sites. Brain remodelling also included structures that are not directly involved in proprioception, for example, the facial nerve, which was rerouted to a posterior position on the side ipsilateral to CCI (to Bregma −11.4), possibly as a result of the asymmetric expansion and/or contraction of other brain regions.

**Figure 6:**
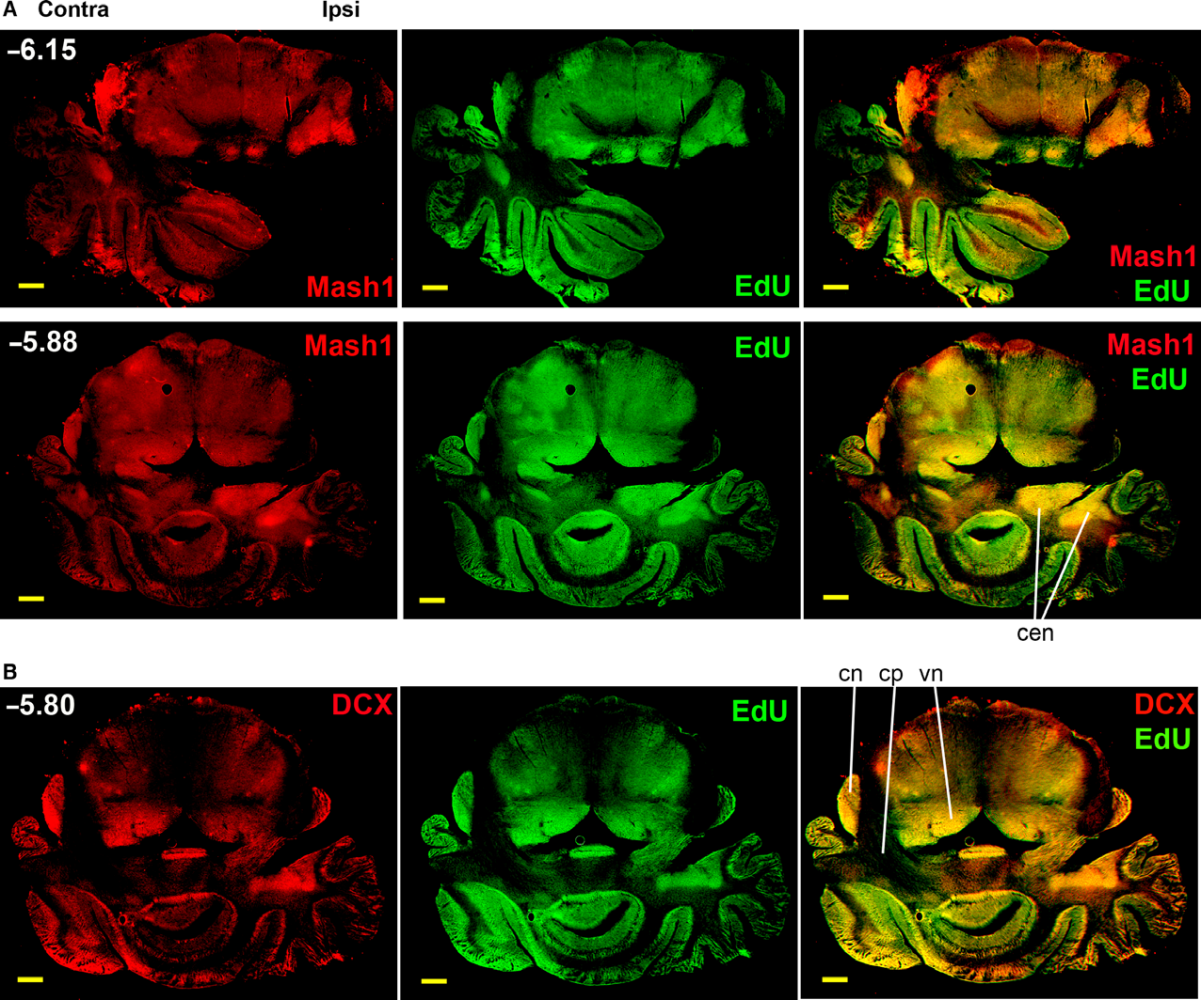
Remodeling of mouse brain 6 weeks post‐CCI is similar to rat. EdU incorporation overlaps with the immunofluorescence detection of neurogenic markers Mash1 (**A**) and DCX (**B**). Intensely stained areas underwent resizing, for example, the cerebellar lobules, cochlear nuclei (cn) and vestibular nuclei (vn) expanded contralaterally and decreased or disappeared ipsilaterally, suggesting the functional predominance of the contralateral side, while some cerebellar nuclei (cen) increased ipsilaterally, scale bars: 1 mm.

The CCI induced similar anatomical changes in mice, rapidly nuclei enlarging contralateral cerebellar peduncles, vestibular and cochlearn and eliminating these structures ipsilaterally, to the extent that the cerebellum became completely disconnected from the pons ipsilaterally (Fig. 6). Proliferating cells can be detected using the DNA incorporation of thymidine analogue EdU, which can be chemically linked to a fluorescent dye 38, 48. Costaining for cell proliferation marker EdU and neurogenic markers, Mash1 (Fig. 6A) and DCX (Fig. 6B), revealed overlapping areas, likely characterized by active neurogenesis. While Mash1 is a marker for proliferating NPCs, DCX is expressed in post‐mitotic immature neurons 49. EdU staining is cumulative, indicating all the cells that transited a proliferative stage during the 3‐day exposure to EdU, while neurogenic markers are limited to transient stages of neuronal differentiation, therefore these two types of staining do not completely overlap. The increased staining of contralateral relative to ipsilateral brain structures, such as the cochlear (cn) and vestibular (vn) nuclei is clearly visible.

### Interference with adult neurogenesis prevents CCI‐induced brain remodelling

**Figure 7:**
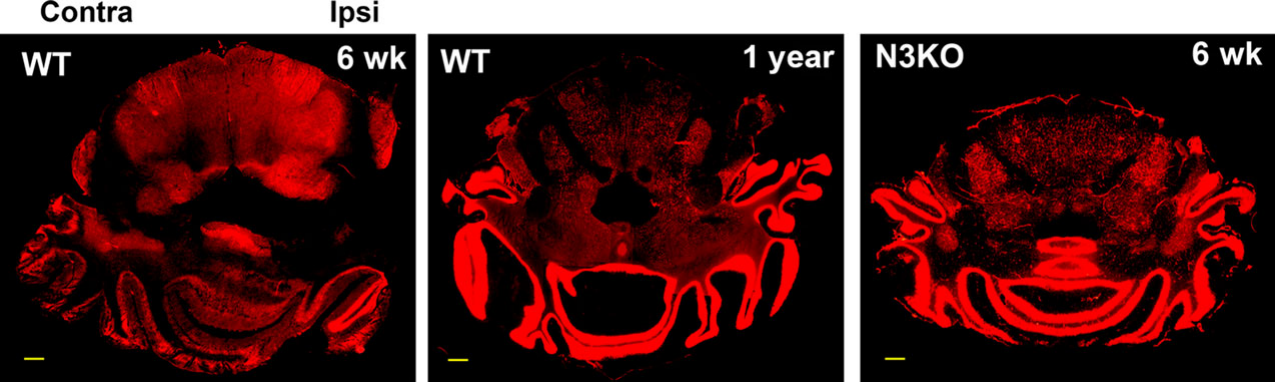
CCI‐induced remodelling of mouse brain is absent in Notch3 KO mice. Post‐CCI brain reorganization in wild‐type mouse (WT), visualized by NeuN staining, shows dramatic changes 6 weeks post‐CCI, resulting in the elimination of the ipsilateral cerebellar peduncles and the disconnection of cerebellum from the pons, as seen in Figure 6. The symmetry of brain organization is partially restored 1 year post‐CCI. In contrast, Notch3 KO mice do not show any sign of structural plasticity 6 weeks post‐CCI (N3KO), Scale bars: 1 mm.

A key role in the regulation of neurogenesis, both during embryonic development and in the adult, is played by the Notch pathway 47, with a distinct role played by Notch3 during adult neurogenesis 38. Notch3 KO mice have impaired neuronal differentiation, resulting in constitutively higher nociceptive sensitivity 38. The anatomical changes observed 6 weeks post CCI in WT mice were absent in Notch3 KO mice (Fig. 7). This suggests that interference with the adult neurogenic process prevents the macroscopic structural changes observed in WT rodents. Similar to rats, the structural changes in WT mice were partially reversed 1 year later, supporting the presence of continuous brain remodelling, resulting from a regain of ipsilateral locomotor function.

### Neurogenic markers are expressed in normal rodent cerebellum

**Figure 8:**
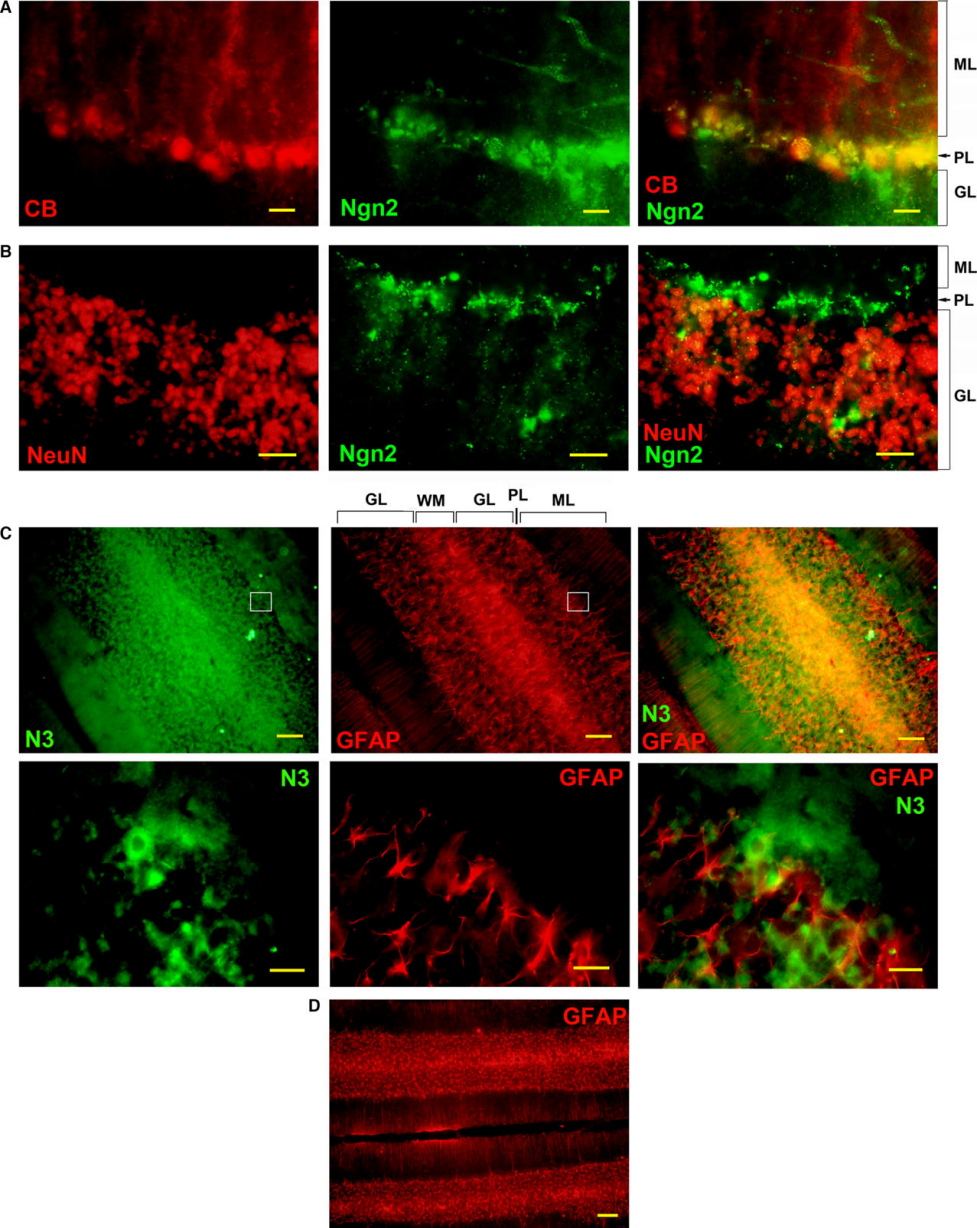
Expression of neurogenic markers in normal rat cerebellum. (**A**) Ngn2 is expressed in the Purkinje layer (PL) in normal rats. CB staining outlines the PL and the molecular layer (ML). The overlap of CB and Ngn2 expression in some Purkinje cell bodies suggests that these neurons are newly generated in the adult. (**B**) NeuN staining outlines the granule cell layer (GL), and is bordered by Ngn2 staining corresponding to the PL. (**C**) Neurogenic marker Notch3 (N3) is expressed in the cerebellar white matter (WM) and in the GL, overlapping with GFAP expression. Notch3 and GFAP do not co‐localize, but are expressed in adjacent cells (detail). Notch3 is expressed in cells with a neuronal phenotype (arrow), while GFAP is expressed in astroglia (arrowhead). (**D**) GFAP expression pattern in CCI rats is identical to its expression in control rats. Scale bars: 100 μm (**B**,** C**), 200 μm (**D**), 20 μm (**A**,** C**‐detail).

The rapid and substantial CCI‐mediated changes in brain anatomy suggested that a large NPC population may pre‐exist in the cerebellum under normal conditions, and that a basal level of neurogenesis is likely continuously present. Otherwise, if starting from a limited, quiescent NPC population, such dramatic changes would require a longer time to develop. Therefore, we tested the presence of neurogenic markers in normal adult rats. We find that Ngn2 expression was present in the cerebellar PL of normal rats (PL, Fig. 8A), overlapping with CB expression in Purkinje neurons, as suggested by Figure 5A. In contrast, there is little overlap between NeuN staining, which characterizes the GL, and Ngn2 staining, which is mostly limited to the PL layer (Fig. 8B). This suggests that Purkinje neurons are continuously regenerated from progenitor cells in the adult under normal conditions, while such regeneration is less active in granule neurons. This observation correlates with the finding that Purkinje neurons are leading the reorganization of cerebellar lobules after CCI, shown in Figure 3. Notch3, a regulator of neurogenesis and neuronal differentiation, is also expressed in the cerebellar lobules, with the highest concentration in the WM, and gradually decreasing into the GL (Fig. 8C). This expression pattern overlaps with the GFAP expression gradient, a marker for reactive astrocytes, which may act as NPCs 50. However, Notch3 and GFAP do not colocalize, but are expressed in adjacent cells (Fig. 8C, detail), as expected, since Notch3 is a marker for cells in the process of acquiring a neuronal phenotype 38. This expression pattern supports the idea that GFAP+‐activated astrocytes in the cerebellar WM may act as neural stem cells and generate new neurons in the adult *via* Notch3+ NPCs. In contrast to the dramatic increase in GFAP expression in the spinal cords of CCI rats 31, related to inflammation, the GFAP expression pattern in the brains of CCI rats (Fig. 8D) is identical to its expression in control animals, indicating that inflammation‐induced gliosis does not contribute to adult brain neurogenesis. Figure 8C and D also show GFAP‐stained parallel filaments extending into the ML, a pattern typical for Bergman glia 51.

## Discussion

### CCI‐induced anatomical changes in the brain are mediated by adult neurogenesis

Here, we have shown that chronic constriction injury of the sciatic nerve results in large‐scale hindbrain remodelling through adult neurogenesis. The implication of adult neurogenesis in adaptive behaviours 52 and in neuronal regeneration 10 has been known for decades. However, although significant structural plasticity has been identified in the human brain using non‐invasive imaging, such changes occurring outside of the generally accepted neurogenic niches (SVZ, DG) have been generally considered a result of neuronal plasticity and/or glial hypertrophy. Our results challenge this idea, suggesting that chronic neuropathic pain induces extensive adult neurogenesis in the rodent hindbrain. We show that unilateral CCI induces large‐scale asymmetric anatomical changes in the cerebellum and pons, which can only be explained by the generation of new neurons. Using a combination of chemical detection and overlapping sequential neuronal markers, we demonstrate the presence in the brain of a continuum of neuronal differentiation states, from proliferating neural stem cells to new fully differentiated neurons, similar to that observed in the spinal cord 38. Over a short period of time, these intermediate progenitors and adult‐generated neurons accumulate in the cerebellum and pons, leading to profound structural changes in regions involved in nociceptive, motor, vestibular, proprioceptive, auditory and gustatory processing, which have not been previously known as neurogenic. These long‐lasting structural changes are an intrinsic indication of the survival and functional integration of the newly generated neurons into existing neural networks. The timing and magnitude of this structural remodelling correlates with similar anatomical changes in the spinal cord 31 and with long‐term behavioural changes described previously 30, and is consistent with the integration of adult‐generated neurons.

Our findings correlate with the increased expression of neurogenesis‐ and apoptosis‐related mRNAs in the cerebellum under normal conditions, including Mash1/Ascl1 and Notch2 53, which suggest an ongoing cerebellar neuronal renewal process similar to the hippocampus. Adult neurogenesis may occur in the cerebellum through the gradual differentiation of astrocytes located in the WM of cerebellar lobules, into neural progenitor cells. The expression of Notch3, a NPC marker, is highest in the WM and gradually declines into the granule layer, suggesting, along with Mash1 and Ngn2 expression, a steady‐state NPC production. Bergmann glia, a type of radial glia which extend parallel processes into the ML, are known to play a role in embryonic neurogenesis 51. Their continued presence through adulthood may reflect a potential role in adult neurogenesis.

### Brain remodelling – an adaptation to changing sensory inputs

The magnitude and long‐lasting effect of the observed anatomical changes, including an obviously asymmetric distribution of neurons, cannot be explained as changes in receptor fields or in glial activation and/or migration, supporting a role for adult neurogenesis. In the spinal cord, a similar CCI‐induced structural plasticity 31 may result from an initial local inflammatory process, inducive of gliosis 54, followed by the differentiation of reactive astrocytes into neural progenitor cells 50. However, in the hindbrain we did not find any signs of CCI‐induced gliosis. Alternatively, the hindbrain could normally undergo a continuous process of slow neuronal regeneration, which would allow accelerated remodelling under changing sensory inputs, similar to the adaptive role of the hippocampus 55. Such basal neurogenic process may be accelerated by a shift in sensory input to one side of the brain, and inhibited by a reduction in sensory input to the other side, as an adaptive mechanism, resulting in the dramatic anatomical changes observed. Peripheral nerve injury is thus processed in the cerebellum as a multisensory perception 56, 57. Chronic nociceptive stimuli inhibit motor output through feedback from higher processing centres in the brain 58. Direct nociceptive input to the cerebellum is also likely to play an important role 33, 34. As a result, rodents subjected to CCI undergo significant long‐term posture changes, shifting the balance to the contralateral side to avoid using the injured leg. Conceivably, this would induce compensatory changes in proprioceptive and vestibular sensory inputs, as well as in visual cues 59, resulting in large‐scale asymmetrical structural changes. In turn, the magnitude of these anatomical modifications may indirectly affect regions involved in other sensory modalities, such as auditory and gustatory, as well as in affective processing. A basal level of continuous neurogenesis would explain these rapid, large‐scale structural changes.

### Cerebellar rearrangements may represent a form of proprioceptive and vestibular memory

The continuous production of hindbrain, including cerebellar, neurons throughout adult life might reflect a proprioceptive and vestibular memory analogous to the olfactory memory 60 and the nociceptive memory 31. This may underlie an experience‐dependent adaptation, which is typical for other established neurogenic niches in the brain 55. Since an individual is exposed to most sensory inputs only after birth, including posture and balance against gravity, this may explain the protracted post‐natal development of the cerebellum relative to other parts of the brain. This sensory memory could emerge from development adapted and fine‐tuned to an average stimulus intensity level to which the organism is normally exposed, for example, to a vertical and bilaterally symmetric posture. Transient changes in sensory input, such as transient changes in position, would be compared to the ‘memorized’ basal value of the stimulus and contribute to the continuous readjustment of posture and balance in relation to external stimuli. However, chronic changes in basal stimulus intensity, such as chronic posture changes after unilateral CCI, would shift the basal perception level permanently to a new value, by asymmetrically influencing the neuron turnover rate, representing in fact a sensory adaptation phenomenon. The partial restoration of hindbrain anatomy back to its original state after the elimination of the asymmetrical external stimulus is an additional demonstration of a continuous neuron turnover leading to a restoration to the original basal stimulus value. However, the restoration process is much slower than the initial response to pain, reflecting a long‐lasting proprioceptive/vestibular memory as mentioned above.

### Clinical implications of hindbrain remodelling

The potential translation of these findings to human brain plasticity remains to be determined. Human adult neurogenesis has unique characteristics in comparison with other mammals. For example, adult neurogenesis in the olfactory bulb, well‐established in other mammals, is virtually absent in humans 61. This is, however, not unexpected, since in humans the perception of smell has lost much of the importance it maintains in most other mammals. However, proprioception and balance maintain in humans the same importance as in other mammals. In addition to sensory and motor control, the cerebellum is also involved in emotion processing 62. Therefore, it is intriguing to speculate that the findings described here may have implications for clinical aspects such as chronic pain, cross‐modal sensory perception, neuropsychology, psychiatric disorders as well as central nervous system (CNS) regeneration from injury or disease.

Hindbrain remodelling was still noticeable 1 year post CCI, while corresponding spinal cord structural changes became undetectable within 6 months 31 and the behavioural changes, such as increased nociceptive susceptibility, have been shown to revert to normal within 3 months 30. This implies that the changes in sensory modalities complementary to nociception could be more persistent than the primary nociceptive stimulus, which may explain some clinical manifestations of chronic pain in the absence of underlying nerve injury. Hindbrain remodelling also affected regions involved in unrelated sensory modalities, for example, the cochlear and olivary nuclei (auditory) and the facial nerve (gustatory). Thus, cross‐modal sensory interactions may emerge directly from structural rearrangements at intermediate levels, such as the cerebellum, in addition to higher cortical processing. Future studies will determine whether other sensory modalities, such as hearing, are indeed altered asymmetrically after CCI, as suggested by hindbrain remodelling. This would be an additional indication that newly generated neurons integrate functionally into existing sensory circuits.

It is interesting to note that some cerebellar disorders, such as inherited ataxias, result from mutations in proteins involved in neurogenesis and/or synaptic connectivity 63. An example is cochlin, involved in stem cell self‐renewal 64. Certainly, in such genetic disorders it is often difficult to separate the defects in embryonic development from those of adult neurogenesis. However, since some ataxia symptoms begin later in life, in older children or even in adults, it is likely that impaired adult neurogenesis plays the major role. The fact that cerebellar adult neurogenesis has escaped detection until now suggests that the neuronal renewal rate, including both neuronal death and neurogenesis, is very low under normal conditions. Still, in the absence of active neurogenesis, programmed neuronal death, albeit slow, gradually leads to a loss of function. As a result, many cerebellar disorders are considered to be neurodegenerative, and stem cell therapy was proposed as a treatment 65. However, an alternative treatment may be the use of growth factors and other functionally similar small molecules, which stimulate endogenous neurogenesis and delay neuronal death at the same time 66, 67.

While in the experiments described here, animals were forced to shift balance, posture and motor activity to the side contralateral to injury, it would be interesting to determine what changes occur in the cerebellum in the absence of any balance or motor stimulus, for example, in animals or humans exposed to long periods of weightlessness. It is reasonable to speculate that a lack of gravity would result in reduced cerebellar neurogenesis, possibly leading to a contraction of cerebellar lobules and also impacting affective and cognitive processes. Surprisingly, a similar process also occurs under hypergravity 68, however, the mechanism could be different, for example, an increase in neurodegeneration, rather than a decrease in neurogenesis. It would be important to determine whether any such changes are reversible upon restoration of gravity, similar to the reversal shown here after restoration of normal posture. The reversibility of such structural and functional changes would be another indication that the newly generated neurons can integrate functionally into existing cerebellar circuits and restore normal function.

In conclusion, our study provides evidence that chronic neuropathic pain induces long‐term, large‐scale hindbrain anatomical rearrangements, potentially as an indirect effect due to the associated posture changes. The scale of these rearrangements and the presence of neurogenic markers suggest that hindbrain regeneration and remodelling occur continuously even under normal conditions, possibly as an adaptive response in addition to neuronal plasticity.

## Conflict of interest

There are no potential conflicts of interest.

## Author contribution

GR: conception, design, data acquisition, manuscript writing; GR and JM: data analysis.

## Supporting information


**Figure S1** Cluster of CB+ cells at the growing tip of the cerebellar lobule. The tip of the growing cerebellar lobule contains a cluster of CB+ cells. Further away from the tip of the lobule, these cells become organized in two parallel layers corresponding to Purkinje neurons, scale bar: 200 μm.
