# Supplementary figures and images for: Peripheral nerve injury induces adult brain neurogenesis and remodelling
Source: J Cell Mol Med. 2016 Sep 24;21(2):299–314. doi: 10.1111/jcmm.12965 (PMC5264155; doi:10.1111/jcmm.12965)

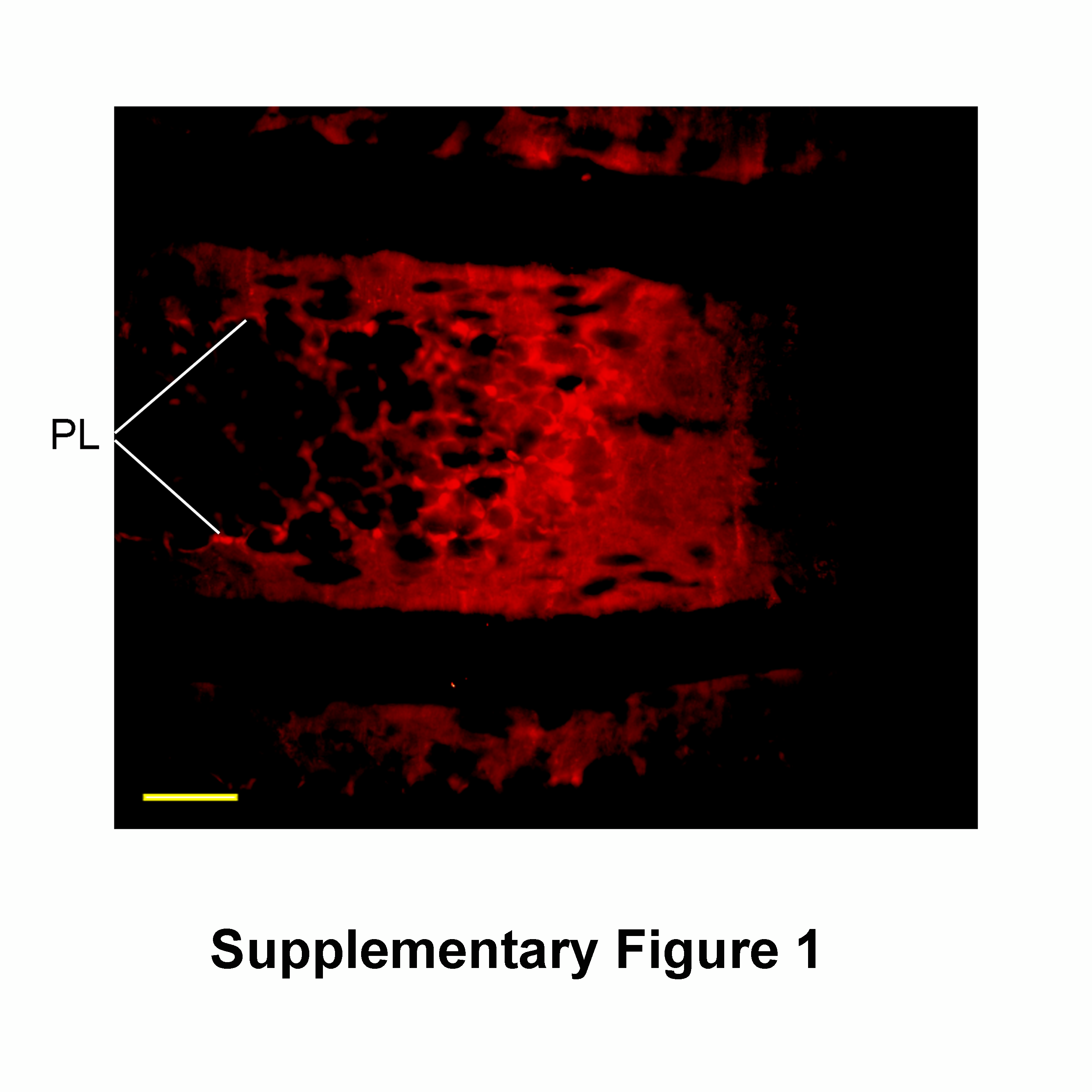

Supplement: Supplementary file 1 — Figure S1 Cluster of CB+ cells at the growing tip of the cerebellar lobule. The tip of the growing cerebellar lobule contains a cluster of CB+ cells. Further away from the tip of the lobule, these cells become organized in two parallel layers corresponding to Purkinje neurons, scale bar: 200 μm. [file JCMM-21-299-s001.tif]
